# Treatment of breast cancer 2 (BRCA2)-mutant follicular dendritic cell sarcoma with a poly ADP-ribose polymerase (PARP) inhibitor: a case report

**DOI:** 10.1186/s13104-016-2189-x

**Published:** 2016-08-03

**Authors:** Charlotte R. Lemech, Rachel Williams, Stephen R. Thompson, Brian McCaughan, Melvin Chin

**Affiliations:** 1 Scientia Clinical Research, UNSW, Sydney, NSW 2052 Australia; 2 Faculty of Medicine, University of New South Wales, Sydney, NSW 2052 Australia; 3 Prince of Wales Hospital, Barker Street, Randwick, 2031 Australia; 4 Royal Prince Alfred Medical Centre, 203/100 Carillon Avenue, Newtown, NSW 2042 Australia

**Keywords:** Follicular dendritic cell sarcoma, BRCA2 mutation, Veliparib, Case report

## Abstract

**Background:**

Follicular dendritic cell sarcoma is a rare tumour with clinical behaviour covering a spectrum from indolent to aggressive disease. Treatment recommendations are currently based on case reports and small series describing combinations of surgery, chemotherapy and radiotherapy providing the best patient outcomes. Recent knowledge on molecular aberrations in this disease have not yet impacted on therapeutic decisions.

**Case presentation:**

We describe a case of progressive follicular dendritic cell sarcoma of the lung and pleura, treated based on knowledge of the tumour’s molecular aberrations. The patient was initially treated with surgery, chemotherapy and radiotherapy and developed disease progression. Mutation testing by Caris molecular intelligence demonstrated a breast cancer 2 gene mutation and further treatment with carboplatin and veliparib achieved disease stabilisation.

**Conclusion:**

Understanding of the molecular profile of rare tumours is key to improve therapeutic decision making and patient outcomes.

## Background

Follicular dendritic cell sarcoma (FDCS) is an uncommon neoplasm of mesenchymal stem cell origin whose clinical course displays much variability. It was first described in 1986 by Monda et al. [1] and was since classified in the 2008 World Health Organization classification of haematolymphoid neoplasms with tumours of histiocytes and dendritic cells, alongside other dendritic cell tumours including Langerhans cell tumours, interdigitating dendritic cell sarcoma and other rare tumours. Follicular dendritic cells (FDC) form a tight meshwork in primary and secondary lymphoid follicles and interact with T and B lymphocytes through antigen presentation and generation and regulation of the germinal centre reaction [2, 3].

FDCS was thought to be an indolent disease and in some patients with localized tumours, prognosis can be favourable with 2 year mortality only 3 % [4]. However some patients have a more aggressive tumour with local recurrence occurring in up to 43 %, distant metastases in 21–24 % and disease-related mortality in 13–17 % [5–7]. A surveillance, epidemiology and end results (SEER) analysis of 54 patients reported a median overall survival of 48 months for those with distant disease [8]. FDCS most commonly presents as nodal disease though extranodal disease is reported in 33–46 % of patients and can involve the bowel, oropharynx, liver, spleen, pancreas, peritoneum, pleura, lung and thyroid [2, 9, 10].

Diagnosis is based on morphological and particularly immunohistochemical appearance with one or more of the following being positive: CD21, CD23, CD35, podoplanin and CXCL-13 [5, 11, 12]. Other FDC markers such as R4/23, Ki-M4, Ki-M4p, Ki-FDC1 may also be positive as well as vimentin, CD68, S100, fascin but these may be nonspecific. Typically, neoplastic cells lack CD1a, desmin and CD45 staining. Between 18.6 and 58 % have been reported as misdiagnosed at initial diagnosis, particularly as the histological appearance may resemble other tumours and FDC markers are not always routine in immunohistochemical analysis [4, 5, 10].

Surgical excision is the standard minimum treatment and intraabdominal tumours and those with other adverse histologic factors should be considered for adjuvant treatment. Overall, better outcomes are reported in those patients whose initial treatment includes a combination of surgery, chemotherapy and radiation where appropriate [5, 6]. Chemotherapy regimens that have documented responses include lymphoma protocols with cyclophosphamide, doxorubicin, vincristine and prednisone (CHOP) and rituximab with CHOP (R-CHOP) as well as sarcoma protocols of doxorubicin and ifosfamide or gemcitabine and a taxane [6].

Due to its rare presentation, molecular analysis has been limited until more recently. Go et al. [13] have reported v-Raf murine sarcoma viral oncogene homolog B (BRAF)^V600E^ mutations in 5 from 27 (18.5 %) cases tested. Phosphatase and tension homolog (PTEN), RET and TP53 mutations have also been documented in FDCS presenting as a thyroid mass [10]. The PTEN and TP53 mutations were similar to those previously described in other malignancies, though the functional significance of the RET mutation was unknown. Griffin et al. [14] have now performed targeted genomic sequencing of 309 known cancer-associated genes in 13 cases of FDCS and found recurrent alterations in nuclear factor (NF)-κB regulatory genes. However, none of these findings have as yet translated into use or trials of molecularly targeted therapies and research in this regard is of course limited by its rarity.

We report the first case using molecularly targeted therapy in combination with chemotherapy in a case of FDCS.

## Case presentation

The patient is a 73 year old Caucasian lady who presented in November 2013 with a hoarse voice. She had a past history of a left total knee replacement, cholecycstectomy and appendecectomy and was on no regular medications. She had a 40 pack year smoking history. There were no significant findings on physical examination, notably no palpable lymphadenopathy, chest nor abdominal findings. CT and FDG-PET imaging demonstrated lesions adjacent to the thoracic aortic arch, aortopulmonary lymphadenopathy, a left superior internal mammary lymph node and a left pleural/para-vertebral lesion near T10. An endobronchial ultrasound (EBUS) guided FNA biopsy of a subcarinal lymph node showed aggregates of epithelioid histiocytes consistent with granulomas without evidence of malignancy. Core biopsy was also attempted and showed abnormal lymphoid cells but without monoclonality. In January 2014, the mediastinal mass, mediastinal lymph node and left pleural mass were resected via a left lateral thoracotomy and talc pleurodesis was performed to debulk the disease and obtain tissue for diagnosis. A timeline of the patient’s clinical course is outlined in Table 1.

**Table 1:** Patient’s clinical course

November 2013	January 2014	February–April 2014	April 2014	June–July 2014
Presented with hoarse voice	Tumour resection and diagnosis of FDCS	CHOP chemotherapy	BRCA2 mutation detected	Mediastinal radiotherapy

April 2015	June 2015	July–October 2015	October 2015	February 2016
Disease recurrence	Carboplatin chemotherapy	Concurrent veliparib with carboplatin	Maintenance veliparib	Stable at last follow-up

The histopathological and immunohistochemical analysis led to a diagnosis of FDCS though the specimen was difficult to interpret due to admixed reactive populations of lymphocytes, fibroblasts, histiocytes and blood vessels. Focally, the tumour expressed some histiocytic and muscle markers, raising the possibility of inflammatory myofibroblastic sarcoma or histiocytic sarcoma but there were areas of bland spindle cells expressing CD21 favouring the diagnosis of FDCS. Interestingly, the tumour was positive for CD21 but negative for CD23 and CD35. Subsequent FDG-PET scan showed residual mediastinal disease, a new T9 metastasis, and pleural-based uptake thought to be secondary to pleurodesis. The case was reviewed at a multidisciplinary team meeting and treatment with CHOP chemotherapy, plus or minus radiotherapy, was recommended. She completed 4 cycles of CHOP and proceeded to mediastinal radiotherapy 50.4 Gy in 28 fractions, encompassing all sites of disease and completed in July 2014.

At the same time, mutation testing with Caris Molecular Intelligence was performed. This is a commercially available platform that identifies a spectrum of clinically actionable treatment options based on immunohistochemistry (IHC), chromogenic and fluorescence in situ hybridization (CISH/FISH), 46- and 592-gene next-generation sequencing (NGS), Sanger sequencing, pyrosequencing and fragment analysis, coupled with a literature review to correlate biomarkers with treatment response. An E1493 fs *BRCA2* mutation was demonstrated and confirmed as a germline mutation. Further family history detailed that her daughter had triple negative breast cancer at age 48 years, a maternal aunt died of postmenopausal breast cancer and this aunt’s own daughter also died of postmenopausal breast cancer. She has paternal German ancestry but no known Jewish ancestry. A hereditary genetics clinic was consulted and risk reducing surgery as well as family counselling was offered.

Follow-up PET in October 2014 demonstrated increased uptake in the left upper and lower lobes and increased intensity in pleural based disease. These changes were thought to be either treatment related or disease progression, with a further PET in February 2015 confirming the latter.

Due to the finding of a *BRCA2* mutation, it was decided to treat with the combination of carboplatin and a poly ADP-ribose polymerase (PARP) inhibitor. A compassionate supply of veliparib was sought. Single agent carboplatin was given for 2 cycles *in June 2015* with the introduction of veliparib 100 mg twice daily at the 3rd cycle *in July 2015*. FDG-PET scan after the 4th cycle showed reduced PET activity in the previously identified lesions. *She completed 6 cycles of carboplatin with concurrent veliparib, followed by maintenance veliparib at 300* *mg twice daily from October 2015.* Her disease remains clinically stable after 5 months of veliparib monotherapy.

Interestingly, when excision repair cross-complementation group 1 (ERCC1) IHC was performed retrospectively, it was negative, consistent with the benefit derived from carboplatin.

## Conclusions

FDCS is a rare tumour with a spectrum of clinical behaviour. While some reports describe an indolent disease [4], others report higher rates of recurrence, metastases and mortality [5, 7]. There are a number of pathological features associated with a poorer prognosis, including size ≥6 cm, coagulative necrosis, high mitotic counts (≥5 per 10 high power field), significant cytologic atypia, younger age and abdominal involvement [5, 6, 15]. As molecular information becomes more readily available for this tumour type, it will also be important to determine its correlation with natural history and therapeutic significance.

This case report describes the use of a PARP inhibitor in combination with carboplatin in the treatment of a FDCS with a *BRCA2* mutation. This is the first report of FDCS with a *BRCA2* mutation and also the first report of the use of molecularly targeted therapy in this disease. *BRCA2* is a tumour suppressor gene involved in deoxyribonucleic (DNA) repair via homologous recombination [16]. Female carriers of a mutation have a 45 and 20 % lifetime risk of breast cancer and ovarian cancer respectively [17]. *BRCA2* mutation is also associated with prostate and pancreatic cancers and melanoma [17]. PARP inhibition in patients with a BRCA mutation demonstrates the concept of synthetic lethality [18]. PARP1 is a DNA repair enzyme that repairs DNA single strand breaks. Thus in cancer cells with *BRCA2* mutation that already display a deficiency in DNA repair, inhibition of PARP1 activity leads to an accumulation of single strand breaks that are converted to double strand breaks that can not be repaired and result in cell death. Veliparib is a small molecular inhibitor of PARP 1 and PARP2 [19] with some activity, though less than olaparib, in so-called PARP trapping, whereby complexes are formed with PARP1 and PARP2 that are also toxic to the cell [20]. There is also preclinical evidence that it potentiates the activity of cisplatin, carboplatin, temozolomide and cyclophosphamide [19, 21]. Veliparib has demonstrated activity in phase I and II studies in BRCA 1/2 mutated ovarian [22] and breast cancers [23] both as a single agent and in combination with carboplatin [24, 25] as well as in patients with brain metastases in combination with whole brain irradiation [26]. In this FDCS case, activity is seen both in the setting of a BRCA2 mutation as well as activity in combination with carboplatin.

The finding of ERCC1 IHC negativity is also of interest here. Although measured in retrospect, it supported the use of a platinum agent as opposed to further lymphoma or sarcoma protocols such as doxorubicin/ifosfamide or gemcitabine/taxane. ERCC1 is a DNA excision repair protein that recognizes and removes cisplatin-induced DNA adducts such that patients with ERCC1-negative tumours derive greater benefit from platinum-based chemotherapy [27]. Thus, with the identification of both a BRCA2 mutation and ERCC1 negativity, this case clearly demonstrates the benefit of molecular profiling in optimising therapy for this rare tumour and avoiding aggressive protocols from which the patient was unlikely to derive benefit.

The study of rare tumours, knowledge on their molecular aberrations and research into therapeutic strategies has been an area of need and is a particular challenge. Interestingly, as genomic alterations across cancer types are described through data repositories such as the International Cancer Genome Consortium, Cancer Genome Project and Cancer Genome Atlas, classification of cancer based on molecular aberrations has gained traction with the molecular signature of some tumours guiding therapeutic decisions rather than the histological subtype. In such a landscape, the challenge lies in attaining evidence for rare tumours to be treated in this manner and novel trial design in this area is key. Alternative strategies such as basket and umbrella studies and Bayesian designs may assist in maximising recruitment and gaining useful information from a smaller sample size. Collaboration with the pharmaceutical industry is also required to gain access to drugs that may have been tested in one tumour type but may apply to rarer cancers with similar genetic aberrations.

This case highlights the importance of molecular profiling across rare tumours both to better understand their biology and provide insight into potential therapeutic strategies. Well-designed multicentre clinical trials in this area with the collaboration of clinicians, researchers, statisticians and pharma is required to provide better outcomes for this patient group.

## Abbreviations

BRCA2: breast cancer 2
PARP: poly ADP-ribose polymerase
FDCS: follicular dendritic cell sarcoma
FDC: follicular dendritic cells
SEER: surveillance, epidemiology and end results
CHOP: cyclophosphamide, doxorubicin, vincristine and prednisone
R-CHOP: cyclophosphamide, doxorubicin, vincristine and prednisone and rituximab
BRAF: v-Raf murine sarcoma viral oncogene homolog B
CISH/FISH: chromogenic/fluorescence in situ hybridization
NGS: next-generation sequencing
PTEN: phosphatase and tension homolog
ERCC1: excision repair cross-complementation group 1
